# Effect of Nitrogen Gas Plasma Generated by a Fast-Pulsed Power Supply Using a Static Induction Thyristor on Scrapie Prion

**DOI:** 10.3390/pathogens9100819

**Published:** 2020-10-06

**Authors:** Akikazu Sakudo, Yuichiro Imanishi, Azumi Hirata, Yuichi Koga, Hideharu Shintani

**Affiliations:** 1 School of Veterinary Medicine, Okayama University of Science, Imabari, Ehime 794-8555, Japan; 2 Laboratory of Biometabolic Chemistry, School of Health Sciences, University of the Ryukyus, Nishihara, Okinawa 903-0215, Japan; 3 NGK Insulators Ltd., Nagoya, Aichi 467-8530, Japan; roy.imanishi56222@gmail.com; 4 Energy Support Corporation, Inuyama, Aichi 484-8505, Japan; 5 Department of Anatomy and Cell Biology, Faculty of Medicine, Osaka Medical College, Takatsuki, Osaka 569-8686, Japan; an1026@osaka-med.ac.jp; 6 Department of Biotechnology, Graduate School of Engineering, Osaka University, Suita, Osaka 565-0871, Japan; kogay@mls.eng.osaka-u.ac.jp; 7 Department of Science and Engineering, Chuo University, Tokyo 112-8551, Japan; hshintani@jcom.zaq.ne.jp

**Keywords:** discharge, gas plasma, inactivation, PMCA, prion, scrapie

## Abstract

Previous studies show that nitrogen gas plasma generated by a fast-pulsed power supply using a static induction thyristor has both virucidal and bactericidal effects. In this study, nitrogen gas plasma was further evaluated for its potential effects on prions, which are well known to be the most resistant pathogen to both chemical and physical inactivation. Aliquots (10 μL) of mouse brain homogenate infected with Chandler scrapie prion were spotted onto cover glasses and subjected to nitrogen gas plasma. Treated samples were recovered and subjected to further analyses. Control prion samples were prepared in exactly the same way but without plasma treatment. Protein misfolding cyclic amplification (PMCA) showed that nitrogen gas plasma treatment at 1.5 kilo pulse per second for 15 or 30 min caused a reduction in the in vitro propagation level of PrPres (proteinase K-resistant prion protein), which was used as an index of abnormal prion protein (PrP^Sc^). Moreover, mice injected with prion treated with plasma for 30 min showed longer survival than mice injected with control prion, indicating that nitrogen gas plasma treatment decreased prion infectivity. Altogether, these results suggest that nitrogen gas plasma treatment can inactivate scrapie prions by decreasing the propagation activity and infectivity of PrP^Sc^.

## 1. Introduction

Proteinaceous infectious particles or “prions” cause transmissible spongiform encephalopathies (TSEs) or prion diseases (PDs), including scrapie in small ruminants, bovine spongiform encephalopathy (BSE) in cattle, and Creutzfeldt–Jakob disease (CJD) in humans [1,2,3]. The major component of prions is an abnormal isoform of prion protein (PrP^Sc^). Conversion of the cellular isoform of prion protein (PrP^C^) to the conformationally refolded, β-sheet-rich isoform PrP^Sc^ is a critical event in the pathogenesis of all prion diseases [3,4,5]. The interaction between PrP^Sc^ and PrP^C^ starts a chain reaction, in which each newly converted PrP^Sc^ molecule interacts with further PrP^C^ molecules, leading to an accumulation of PrP^Sc^ [2,6]. The reaction and propagation of PrP^Sc^ can be mimicked in vitro by protein misfolding cyclic amplification (PMCA) [7,8,9].

Overall, prions are recognized as the most resistant type of pathogen to both chemical and physical inactivation [10]. As a result, an efficient and safe method for inactivating prions is needed. Most importantly, the infectivity of prions is not completely lost by conventional sterilization procedures, such as autoclaving at 121 °C for 20 min or exposure to ultraviolet (UV) or γ-ray irradiation [10,11,12]. In the case of autoclaving, severe conditions (134 °C for 18 min) are required to completely inactivate the infectious prion agent [13]. However, such treatments cannot be used for heat-sensitive medical devices. As an alternative, a hydrogen peroxide gas plasma sterilization device, STERRAD^®^ (Advanced Sterilization Products (ASP), Johnson & Johnson company, Irvine, CA, USA), is often used to sterilize heat-sensitive medical devices [14]. In addition to hydrogen peroxide, oxidizing gases such as peracetic acid [15], ozone (O_3_), and chlorine dioxide (ClO_2_) [16] have been applied to plasma sterilization. However, the US Food and Drug Administration (FDA) has issued a toxicity safety alert for clinical medical devices that have been sterilized by these plasma-based systems [17,18]. Although the microbicidal activity of these systems has been proved, there are potential problems associated with the compatibility of the device and with toxic residues left on the medical instruments [19]. 

Recently, we succeeded in generating nitrogen gas plasma by applying a high-voltage pulse for a short time using an induction energy storage (IES) circuit with a static induction (SI) thyristor to nitrogen using a BLP–TES (bipolar and low-pressure plasma–triple effects sterilization) device (NGK Insulators, Ltd., Nagoya, Japan) [20]. Interestingly, BLP–TES has shown virucidal and bactericidal activity [20,21,22,23,24,25] and been found to be superior to STERRAD^®^ in terms of endotoxin inactivation efficiency [20]. Those findings have prompted us to consider the potential use of this technology in the inactivation of prions. 

Here, we have examined whether nitrogen gas plasma generated by BLP–TES can inactivate scrapie prion. Both PMCA and mouse bioassays were performed to elucidate whether treatment of samples by nitrogen gas plasma using BLP–TES can reduce the propagation activity and infectivity of prions.

## 2. Results

### 2.1. Nitrogen Gas Plasma Treatment of Scrapie Prion Reduced Prion Amplification In Vitro

**Figure 1:**
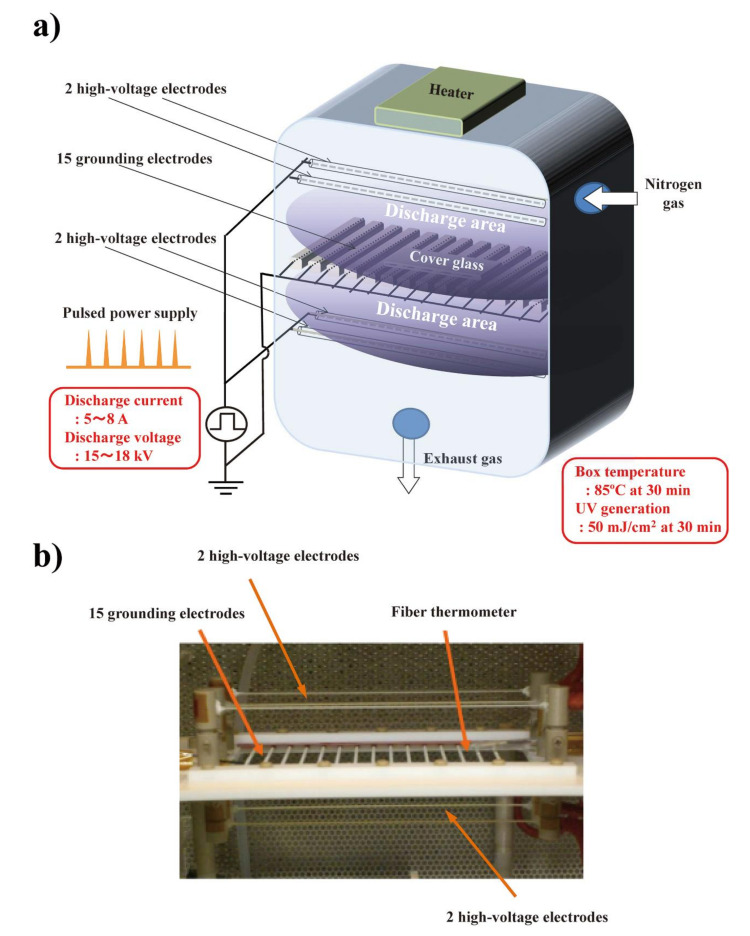
The nitrogen gas plasma instrument, bipolar and low-pressure plasma–triple effects sterilization (BLP–TES) used in this study. (**a**) Schematic illustration of BLP–TES. The 15 grounding electrodes are located centrally between the 2 upper and 2 lower high-voltage electrodes (4 high-voltage electrodes in total). During the electrical discharge at 1.5 kpps (kilo pulse per second), the chamber box was kept at a pressure of 0.5 atm. Next, nitrogen gas (99.9995%; Okano, Okinawa, Japan) was applied at a flow rate of 10 L/min. The discharge current was 5~8 A, while the discharge voltage was 15~18 kV. An aliquot (10 µL) of 10% Chandler scrapie prion-infected mouse brain homogenate was spotted onto a cover glass. The cover glass was then placed on the grounding electrodes during the discharge. After 30 min of electrical discharge, the temperature of the chamber box measured by a fiber thermometer was 85 °C, while UV generation was 50 mJ/cm^2^ [22]. (**b**) Photo image of the high-voltage and grounding electrodes, and the fiber thermometer in BLP–TES.

**Figure 2:**
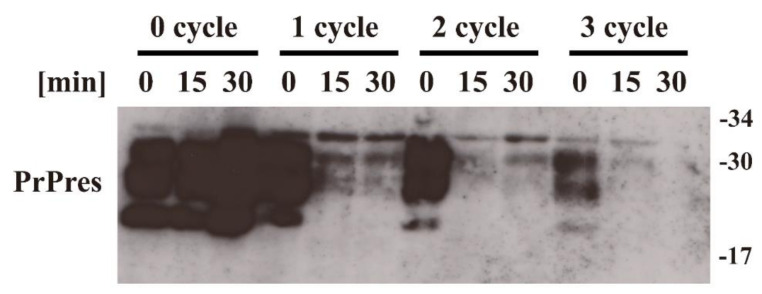
Protein misfolding cyclic amplification (PMCA) showing that the propagation activity of prions is reduced by nitrogen gas plasma treatment. Scrapie prion (Chandler strain) samples treated with nitrogen gas plasma for 0, 15, or 30 min at 1.5 kpps (kilo pulse per second) in a BLP–TES instrument were subjected to PMCA using mouse brain homogenate as a substrate for cellular isoform of prion protein (PrP^C^). After each round of PMCA, the samples were further diluted 1:10 with the PrP^C^ substrate and subjected to another round of PMCA. Samples from before (0 cycle) and from each round of PMCA were treated with proteinase K (PK) to determine the level of PrPres, which is used as an index of the abnormal isoform of prion protein (PrP^Sc^). Samples were analyzed by sodium dodecyl sulfate–polyacrylamide gel electrophoresis (SDS–PAGE) followed by Western blotting using SAF83 anti-PrP antibody. Molecular mass markers (kDa) are indicated on the right-hand side of the gel.

First, we applied the BLP–TES device to the nitrogen gas plasma treatment of prion derived from Chandler scrapie prion-infected mouse brain homogenate. This device generates nitrogen gas plasma by the application of short high-voltage pulses using an SI thyristor (Figure 1). The change in in vitro prion propagation activity due to nitrogen gas plasma treatment at 1.5 kilo pulse per second (kpps) was evaluated by PMCA using C57BL/6J mouse brain homogenates as the PrP^C^ substrate. In total, three rounds of PMCA (1~3 cycles) were performed to test the propagation ability of prion samples treated with nitrogen gas for different durations (0, 15, and 30 min) (Figure 2). Note that the 0-min-treated samples were prepared in exactly the same way as the plasma-treated samples, except that the nitrogen gas plasma was not applied. In each round of PMCA, the samples were diluted 1:10 with the PrP^C^ substrate. The resultant samples were treated with proteinase K (PK) to determine the level of PK-resistant prion protein (PrPres), which is used as an index of PrP^Sc^ [11], and analyzed by sodium dodecyl sulfate–polyacrylamide gel electrophoresis (SDS–PAGE) followed by Western blotting using SAF83 anti-PrP antibody.

The signal for PrPres was clearly detected in the 1st, 2nd, and 3rd cycles of PMCA for the 0-min-treated sample. By contrast, the signal for PrPres was greatly reduced in the 2nd and 3rd cycles of PMCA for the 15-min- and 30-min-treated samples. These observations suggest that nitrogen gas plasma treatment decreases the ability of scrapie prion to proliferate in vitro.

### 2.2. Nitrogen Gas Plasma Treatment Reduced Prion Infectivity in a Mouse Bioassay

**Figure 3:**
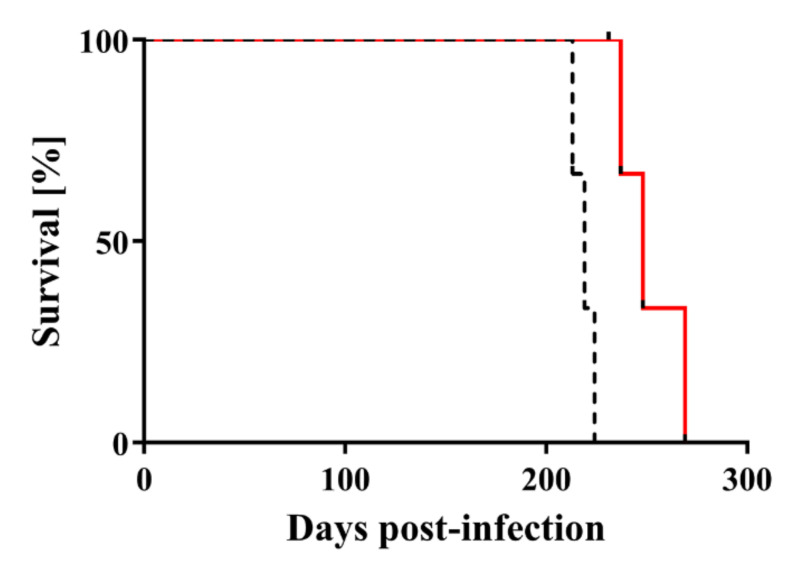
Mouse bioassay shows that the infectivity of scrapie prions is reduced by nitrogen gas plasma treatment. Survival curves are shown for C57BL6/J mice injected intracerebrally with Chandler prion-infected brain homogenate that was treated with nitrogen gas plasma using BLP–TES for 0 min (dotted black line) or for 30 min at 1.5 kpps (red line). Mice injected with nitrogen gas plasma-treated prions (30 min) lived significantly longer than mice injected with 0-min-treated prions (log-rank test, *p* < 0.01).

**Figure 4:**
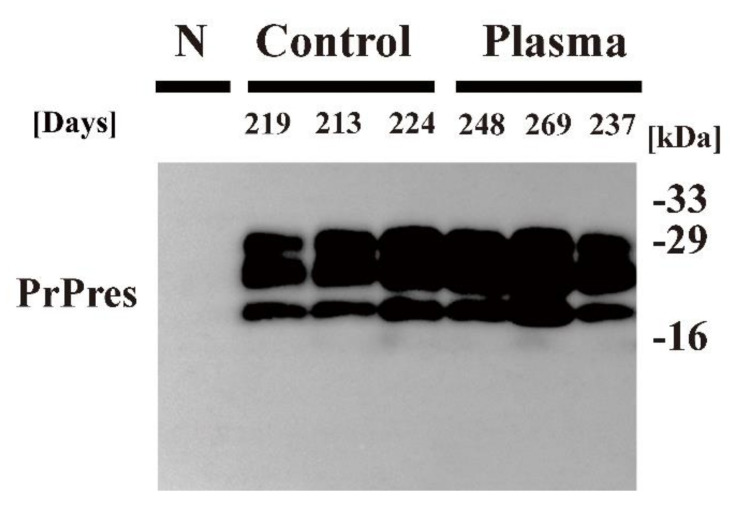
PrP^Sc^ detection in the brain of mice that succumbed to disease. The brain of C57BL6/J mice injected intracerebrally with Chandler prion-infected brain homogenate that was treated with nitrogen gas plasma using BLP–TES for 0 min (Control) or 30 min at 1.5 kpps (Plasma) was collected at the time of end-stage disease (days) when the mice showed clinical symptoms such as tremors and ataxia. Samples were subjected to treatment with PK to determine the presence of PrPres. Samples were analyzed by SDS–PAGE, followed by Western blotting using anti-PrP antibody SAF83. Molecular mass markers (kDa) are indicated on the right-hand side of the gel. N: normal (uninfected) mouse brain.

**Figure 5:**
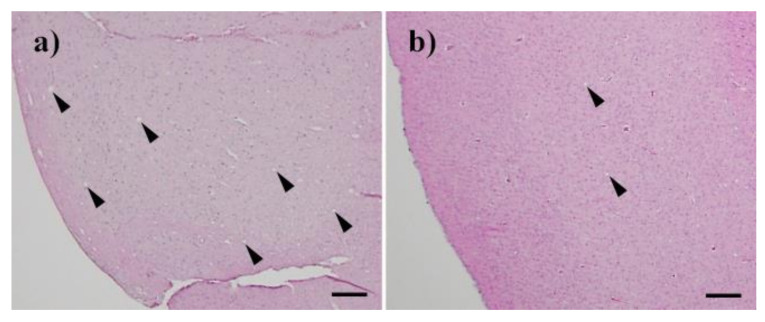
Histopathological changes in the brain of mice that succumbed to disease. H&E (Hematoxylin and eosin) staining showed spongiform changes in the brain of the succumbed mice after injection of Chandler prion treated with nitrogen gas plasma for (**a**) 0 min and (**b**) 30 min. Bar: 100 µm.

**Figure 6:**
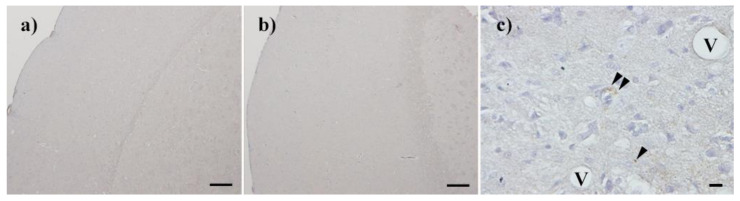
PrP^Sc^ accumulation in the brain of mice that succumbed to disease. Accumulation of PrP^Sc^ was confirmed by immunohistochemistry with SAF83 in the brain of the succumbed mice after injection of Chandler prion treated with nitrogen gas plasma for 0 min (**a**,**c**) and 30 min (**b**). V: Vacuole; Arrowheads: PrP^Sc^ deposits; Bar: 100 µm for (**a**) and (**b**), 10 µm for (**c**).

Next, Chandler scrapie prion that had been treated by nitrogen gas plasma for 30 min at 1.5 kpps or 0-min-treated Chandler scrapie prion was injected intracerebrally into C57BL6/J mice, and the two groups were compared in terms of disease incubation time and survival (Figure 3). All three mice injected with 0-min-treated prion showed abnormal behavior, including tremors and ataxia, within 224 days. By contrast, the three mice injected with prion treated by nitrogen gas plasma (30 min) showed no clinical symptoms of prion disease up to 236 days after injection and survived until 237~269 days. The incubation time was 218.7 ± 3.2 days for mice injected with 0-min-treated prion, and 251.3 ± 9.4 days for mice injected with nitrogen gas plasma-treated prion (Table 1). Western blotting showed that PrPres was clearly detected in all mice that died due to prion disease, including three mice of the control group (injected with prion samples prepared on a cover glass in the same way as nitrogen gas plasma-treated samples but without plasma treatment) and three mice of the group injected with prions treated with nitrogen gas plasma for 30 min (Figure 4). These observations confirmed that all mice died due to prion disease. In addition, vacuolation and PrP^Sc^ accumulation in the brain of representative mice that succumbed to the disease were confirmed by hematoxylin and eosin (H&E) staining and immunohistochemistry, respectively (Table 2). Representative mice in both groups were checked by histopathological analysis. The results confirmed the presence of spongiform changes in the brain of mice that succumbed to disease in both groups (Figure 5). In addition, immunohistochemistry with antibody SAF83 showed that PrP^Sc^ accumulated in the brain of mice that died in both groups (Figure 6). Therefore, these results further confirmed that all mice died due to prion disease.

Next, the survival curves of the groups were compared by statistical analysis. The survival curves reflected a similar trend in terms of incubation time, but there were significant differences between the two groups (*p* < 0.05) (Figure 3). Specifically, log-rank test showed that mice injected with prion treated by nitrogen gas plasma (30 min) lived significantly longer than those injected with 0-min-treated prion. These results indicate that nitrogen gas plasma treatment delays the onset of the clinical signs of disease and prolongs survival. Taken together, these results indicate that nitrogen gas plasma treatment decreases the infectivity of scrapie prion.

## 3. Discussion

The prion agent is an extremely resistant pathogen; thus, the development of prion inactivation methods is important in preventing iatrogenic disease derived from exposure to prion-contaminated medical materials [10]. In this study, we analyzed the effect of nitrogen gas plasma as a potential disinfection method for the scrapie prion agent. PMCA analysis and a mouse bioassay were used to investigate the properties of prions after nitrogen gas plasma treatment using a BLP–TES device. The results showed that nitrogen gas plasma treatment applied at 1.5 kpps for 15 and 30 min reduced the in vitro propagation ability of scrapie prion and reduced the infectivity of prions, respectively.

Previous studies have shown that influenza virus, respiratory syncytial virus, and adenovirus are inactivated within 5 min by BLP–TES applied at 1.5 kpps [23,24,25], whereas treatment for more than 15 min is required for the inactivation of bacterial spores [22]. Overall, these findings are consistent with the notion that prion agent is a more resistant pathogen than viruses or bacteria including bacterial spores. The present results also support the idea that prion is the most resistant pathogen to chemical and physical treatments including nitrogen gas plasma.

The gas plasma sterilization device STERRAD^®^, which can be used for heat-sensitive medical devices, utilizes the oxidizing ability of hydrogen peroxide gas [14,26]. However, that device is not a true plasma sterilizer, because the sterilization factor is hydrogen peroxide gas rather than the plasma. In the STERRAD^®^ device, under the common cycle conditions, the plasma is generated only after exposure and removal of the gas by a vacuum [14]. In addition, no difference in microbicidal activity was observed in the presence and absence of plasma in STERRAD^®^ [27]. Therefore, the observed antimicrobial effect of STERRAD^®^ is due to the hydrogen peroxide gas and not gas plasma.

Over the past 20 years, various studies have reported the microbicidal effect of plasma including inactivation of prions [28]. For example, Jalak et al. reported the disinfection of scrapie prions by corona discharge in open air [29], confirming a decrease in both PrP^Sc^ and PrP^C^ by cell infectivity assay and Western blot analysis. In addition, Baxter et al. reported the treatment of scrapie prion by a radio-frequency (RF) plasma generated from an Ar/O_2_ gas mixture [30]. In their experiments, stainless steel spheres were contaminated with scrapie prion and then treated with the RF plasma for 1 h. A hamster bioassay based on injection of the recovered prion indicated that the Ar/O_2_ RF plasma successfully inactivated scrapie prions. Furthermore, in the European project BIODECON on prion treatment using low-pressure inductively coupled plasma, both mouse and hamster bioassays showed that an Ar-plasma efficiently inactivated prions as compared with a mixed Ar/N_2_ plasma [31].

Although those studies demonstrate the inactivation of prions by exposure to plasma generated by various methods and gas mixtures, there have been no studies on the inactivation of prions by nitrogen gas plasma generated by a pulsed power supply using a SI thyristor. Consequently, the present study is the first to demonstrate that both the propagation activity and infectivity of prions are significantly reduced by treatment with nitrogen gas plasma generated by a pulsed power supply using a SI thyristor in a BLP–TES device.

Our group previously reported that nitrogen gas plasma causes protein denaturation [21,22]. Roth et al. [32] also reported the same phenomenon of protein inactivation due to oxidation. In addition, nitrogen gas plasma causes secondary structural changes in a protein [33]. Specifically, nitrogen gas plasma treatment of bovine serum albumin (BSA) resulted in an increase in α-helix and a decrease in β-sheet content of the protein. Although the mechanism underlying the conformational changes remains unclear, the plasma-induced effects were different from those of heat denaturation, which resulted in a decrease in α-helix and an increase in β-sheet content [33]. Moreover, further studies would be required to assess whether nitrogen gas plasma induces the conformational changes of prions.

Our previous studies have shown that heat, UV radiation, and oxidative stress are generated during operation of the BLP–TES device [21,22,24]; however, reactive chemical species seem to contribute to the main mechanism of inactivation caused by nitrogen gas plasma in the case of bacteria and viruses [21,22,24]. Nevertheless, the main chemical and physical factors contributing to inactivation of prions by nitrogen gas plasma have not been clarified. Thus, it will be necessary to analyze which of these factors contributes to reducing the in vitro propagation ability and infectivity of prions in the future. Moreover, further studies are required to determine which of the reactive chemical species in nitrogen gas plasma generated by BLP–TES cause the inactivation of prions. Research into the effect of important reactive chemical species on the function and structure of prions is also required. To establish the optimal conditions for treatment by nitrogen gas plasma, however, a systematic study on the mechanisms by which the nitrogen gas plasma inactivates prions will be necessary.

The present study has a few limitations. First, because the PMCA condition in the present study was suboptimal and not quantitative, optimized PMCA conditions or quantitative real-time quaking-induced conversion (qRT-QuIC) will be required to further analyze the effect of nitrogen gas plasma on prions. Second, because the bioassay in the present study did not provide quantitative results, the reduction in infectivity caused by nitrogen gas plasma is not clear. Further animal bioassays, including endpoint titration [34] and an incubation time interval assay [35], will be required to understand and establish the reduction in infectivity caused by nitrogen gas plasma treatment.

In recent years, prion diseases have become a focus of research interest as a prototype of neurodegenerative protein misfolding diseases [36,37]. Accumulating evidence suggests that other proteins may follow a similar molecular mechanism of self-perpetuating aggregation and cell-to-cell spreading, both in vitro and in animal models [37,38]. Common neurodegenerative diseases such as Alzheimer’s disease (AD) and Parkinson’s disease (PD) share prion-like transmissibility under specific experimental conditions [39]. The present study shows that nitrogen gas plasma treatment by BLP–TES significantly reduces the propagation of prions, suggesting that a similar treatment might also be effective for inactivation of the aggregated products of neurodegenerative diseases such as AD and PD. However, the effect of nitrogen gas plasma might vary among prions derived from different species and among aggregation-prone proteins associated with neurodegenerative diseases. Further studies are required to establish whether nitrogen gas plasma treatment will be useful for the inactivation of various prions derived from humans and animals, as well as other aggregation-prone proteins.

Taken together, the present study suggests that scrapie prion can be inactivated by nitrogen gas plasma generated by a power supply using a SI thyristor in a BLP–TES device. In a medical setting, however, the plasma disinfection/sterilization method will need to be effective on contaminated prion derived from humans. Previous studies have reported that animal prions may behave differently from human prions in susceptibility to chemical and physical treatment [10]. In addition, a recent study has shown that differences in response to heat treatment may depend on the prion strain [40]; therefore, different prion strains may behave differently in nitrogen gas plasma treatment. Thus, further studies on the treatment of various animal and human prions using nitrogen gas plasma are needed before practical use of this technique.

## 4. Materials and Methods

### 4.1. Nitrogen Gas Plasma Device

Nitrogen gas plasma for treatment of prion samples was generated by a BLP–TES device (NGK Insulators Ltd.), in which a short high-voltage pulse generated by an IES circuit with a SI thyristor is applied to nitrogen gas as described previously [20,21,33]. In brief, a 15 cathode (grounding) electrode was set between the 2 upper and 2 lower anode (high-voltage) electrodes. A 10 μL aliquot of 10% brain homogenate from a terminally diseased ICR (Institute of Cancer Research) mouse intracerebrally injected with scrapie prion (Chandler strain), a kind gift from Professor Motohiro Horiuchi (Hokkaido University), was spotted onto a glass coverslip and put on the grid of the grounding electrode. The nitrogen gas plasma was then generated as follows. The chamber box of the BLP–TES device containing the samples was decompressed and degassed, and nitrogen gas (99.9995%; Okano, Okinawa, Japan) was introduced. The electrical discharge was applied at 1.5 kpps (kilo pulse per second) for 0, 15, or 30 min, during which the pressure in the chamber box was maintained at about 0.5 atmospheres. After exposure to the nitrogen gas plasma, the resultant prion samples on the glass coverslips were recovered and resuspended in phosphate-buffered saline (PBS) for analysis.

### 4.2. PMCA

An automatic cross-ultrasonic protein-activating device (ELESTEIN 070-GOT; Elekon Science Corp., Chiba, Japan) was used for PMCA as reported previously [41] with slight modification. Prion samples were mixed 1:10 with brain homogenates of C57BL/6J mouse (Japan SLC, Inc.) as the PrP^C^ source [10% C57BL6/J mouse brain homogenate in PBS containing complete protease inhibitors (Roche Diagnostics, Mannheim, Germany), 4 mM EDTA, and 1% Triton X-100). The amplification process comprised 40 cycles of sonication (3 s pulse oscillation, repeated five times at 1 s intervals), followed by incubation at 37 °C for 1 h with gentle agitation. The amplified products obtained after the 1st round of PMCA were diluted 1:10 with further PrP^C^ substrate, and a 2nd round of amplification was then performed. This process was repeated as necessary. After PMCA, the samples were subjected to Western blotting as described below.

### 4.3. Western Blot Analysis

First, the protein concentration of samples was measured by using a Bio-Rad DC protein assay kit (Bio-Rad, Hercules, CA, USA). To discriminate PrP^Sc^ from PrP^C^, samples containing 60 μg of protein were incubated with or without PK (20 μg/mL) for 30 min at 37 °C, because only PrP^Sc^ is detected after PK treatment. Next, an equal volume of 2× SDS (sodium dodecyl sulfate) gel-loading buffer (90 mM Tris/HCl (pH 6.8), 10% (v/v) 2-mercaptoethanol, 2% (w/v) SDS, 0.02% bromophenol blue, and 20% (v/v) glycerol) was added, and the reaction was terminated by heating at 100 °C for 10 min. SDS–PAGE (12% gel) was used to separate proteins as described previously [42]. A semidry blotting system (Bio Rad, Cambridge, MA, USA) was then used to transfer the proteins to polyvinylidene difluoride (PVDF) membranes (Amersham Biosciences, Piscataway, NJ, USA). The membranes were blocked with 5% skimmed milk (Wako, Osaka, Japan) for 1 h at room temperature, and then incubated for 1 h at room temperature with SAF83 (anti-PrP antibody; SPI Bio, Montigny le Bretonneux, France) in PBS-T (0.1% Tween20 in PBS) containing 0.5% skimmed milk. The membranes were washed three times with PBS-T for 10 min, incubated with horseradish peroxidase (HRP)-labeled anti-mouse immunoglobulin secondary antibody (Jackson Immunoresearch, West Grove, PA) in PBS-T containing 0.5% skimmed milk for 1 h at room temperature, and then washed three more times with PBS-T for 10 min. Blots were developed with an enhanced chemiluminescence (ECL) reagent (Amersham Bioscience), and the chemiluminescence signal was detected by using Ez-Capture MG (ATTO Corp., Tokyo, Japan).

### 4.4. Prion Inoculation

To measure the change in infectivity after nitrogen gas plasma treatment, prion samples (nitrogen gas plasma-treated (30 min) prion-infected mouse brain homogenate or 0-min-treated prion-infected mouse brain homogenate) were 10-fold diluted in PBS and used to inoculate C57BL6/J mice (Japan SLC, Inc.). A 20 μL aliquot of the inoculant was injected into the cerebral ventricle of mice using a microsyringe as described previously [43,44]. Clinical symptoms such as tremors and ataxia were observed at end-stage disease. The number of animals used in the study was kept to a minimum. The animals were housed according to standard animal care protocols and sacrificed in accordance with the University of the Ryukyus guidelines. All experimental procedures were approved by the Animal Ethics Committee of the University of the Ryukyus (approval No. 6001; approval date 17 March 2015).

### 4.5. Histopathology and Immunohistochemistry

The left hemisphere of the brain was fixed in 4% paraformaldehyde and embedded in paraffin. Serial sections were stained with H&E to evaluate neuropathological changes. After epitope retrieval by autoclaving at 121 °C for 15 min, the sections were immersed in 1 mM HCl and then washed with 3% hydrogen peroxide solution. PrP^Sc^ immunocytochemistry was then performed with monoclonal anti-PrP antibody SAF83, followed by an appropriate secondary antibody (Dako EnVision; Dako Japan Co., Tokyo, Japan). Immunoreactivity was visualized by using a DAB-H_2_O_2_ solution (Nichirei Biosciences, Tokyo, Japan). Sections were then counterstained with H&E and observed by using Fluorescence Microscope (BZ-800; Keyence, Osaka, Japan).

### 4.6. Statistical Analysis

Log-rank test was used to compare the survival curves between groups. Differences between 0- min-treated and nitrogen gas plasma-treated (30 min) samples were assessed by unpaired *t*-test. The analyses were carried out by using GraphPad Prism 7 software version 7.02 (GraphPad Prism Software Inc., La Jolla, CA, USA).

## Figures and Tables

**Table 1:** Disease incubation time among mice injected with prion treated by nitrogen gas plasma (30 min) and 0-min-treated prion (Chandler strain).

Nitrogen Gas Plasma Treatment Time	Mean Incubation Time ± SEM ^1^
0 min	218.7 ± 3.2 days
30 min	251.3 ± 9.4 days ^2^

^1^ SEM, standard error of the mean. ^2^ *p* < 0.05.

**Table 2:** Levels of vacuolation and PrP^Sc^ accumulation in the brain of mice that succumbed to disease.

Nitrogen Gas Plasma Treatment Time	Vacuoles in Brain	PrP^Sc^ Accumulation in Brain
0 min	+++	++
30 min	+	++

Relative levels: +, low; ++, medium; +++, high.

## References

[1] Prusiner S.B. (1998). Prions. Proc. Natl. Acad. Sci. USA.

[2] Prusiner S.B. (1991). Molecular biology of prion diseases. Science.

[3] Sakudo A., Ikuta K. (2009). Prion protein functions and dysfunction in prion diseases. Curr. Med. Chem..

[4] Baral P.K., Yin J., Aguzzi A., James M.N.G. (2019). Transition of the prion protein from a structured cellular form (PrP^C^)) to the infectious scrapie agent (PrP^Sc^). Protein Sci..

[5] Pan K.M., Baldwin M., Nguyen J., Gasset M., Serban A., Groth D., Mehlhorn I., Huang Z., Fletterick R.J., Cohen F.E. (1993). Conversion of alpha-helices into beta-sheets features in the formation of the scrapie prion proteins. Proc. Natl. Acad. Sci. USA.

[6] Cohen F.E., Prusiner S.B. (1998). Pathologic conformations of prion proteins. Annu. Rev. Biochem..

[7] Castilla J., Saa P., Morales R., Abid K., Maundrell K., Soto C. (2006). Protein misfolding cyclic amplification for diagnosis and prion propagation studies. Methods Enzymol..

[8] Soto C., Saborio G.P., Anderes L. (2002). Cyclic amplification of protein misfolding: Application to prion-related disorders and beyond. Trends Neurosci..

[9] Giaccone G., Moda F. (2020). PMCA Applications for prion detection in peripheral tissues of patients with variant Creutzfeldt-Jakob disease. Biomolecules.

[10] Sakudo A. (2020). Inactivation methods for prions. Curr. Issues Mol. Biol..

[11] Sakudo A., Ano Y., Onodera T., Nitta K., Shintani H., Ikuta K., Tanaka Y. (2011). Fundamentals of prions and their inactivation (review). Int J. Mol Med..

[12] Fichet G., Comoy E., Duval C., Antloga K., Dehen C., Charbonnier A., McDonnell G., Brown P., Lasmezas C.I., Deslys J.P. (2004). Novel methods for disinfection of prion-contaminated medical devices. Lancet.

[13] Rutala W.A., Weber D.J. (2010). Society for Healthcare Epidemiology of, A. Guideline for disinfection and sterilization of prion-contaminated medical instruments. Infect. Control. Hosp. Epidemiol..

[14] Jacobs P.T., Lin S.M. (1987). Hydrogen peroxide plasma sterilization system. U.S. Patent.

[15] Moulton K.A., Campbell B.A., Caputo R.A. (1990). Plasma Sterilizing Process with Pulsed Antimicrobial Agent. U.S. Patent.

[16] Finnegan M., Linley E., Denyer S.P., McDonnell G., Simons C., Maillard J.Y. (2010). Mode of action of hydrogen peroxide and other oxidizing agents: Differences between liquid and gas forms. J. Antimicrob. Chemother..

[17] (1998). FDA Talk Paper No.T98-17.

[18] Gibbs J., Matthees S., UBM Americas, UBM plc Company Lessons Learned from the AbTox Ruling. https://www.mddionline.com/lessons-learned-abtox-ruling.

[19] Furuhata S., Nishimura C., Furuhashi N., Miyakawa J., Ozawa M., Usami K., Misawa A., Karasawa H. (2000). Effectiveness test of low temperature plasma sterilization method using peracetic acid and hydrogen peroxide (in Japanese). Jpn. J. Assoc. Op. Med..

[20] Shintani H., Shimizu N., Imanishi Y., Sekiya T., Tamazawa K., Taniguchi A., Kido N. (2007). Inactivation of microorganisms and endotoxins by low temperature nitrogen gas plasma exposure. Biocontrol Sci..

[21] Sakudo A., Misawa T., Shimizu N., Imanishi Y. (2014). N_2_ gas plasma inactivates influenza virus mediated by oxidative stress. Front. Biosci..

[22] Sakudo A., Toyokawa Y., Nakamura T., Yagyu Y., Imanishi Y. (2017). Nitrogen gas plasma treatment of bacterial spores induces oxidative stress that damages the genomic DNA. Mol. Med. Rep..

[23] Sakudo A., Toyokawa Y., Imanishi Y., Murakami T. (2017). Crucial roles of reactive chemical species in modification of respiratory syncytial virus by nitrogen gas plasma. Mater. Sci. Eng. C.

[24] Sakudo A., Toyokawa Y., Imanishi Y. (2016). Nitrogen gas plasma generated by a static induction thyristor as a pulsed power supply inactivates adenovirus. PLoS ONE.

[25] Sakudo A., Shimizu N., Imanishi Y., Ikuta K. (2013). N_2_ gas plasma inactivates influenza virus by inducing changes in viral surface morphology, protein, and genomic RNA. Biomed. Res. Int..

[26] Jacobs P.T., Lin S.M. (1985). Hydrogen Peroxide Plasma Sterilization System. U.S. Patent.

[27] Krebs M.C., Bécasse P., Verjat D., Darbord J.C. (1998). Gas plasma sterilization: Relative efficacy of the hydrogen peroxide phase compared with that of the plasma phase. Int. J. Pharm..

[28] Sakudo A., Yagyu Y., Onodera T. (2019). Disinfection and sterilization using plasma technology: Fundamentals and future perspectives for biological applications. Int. J. Mol. Sci..

[29] Julák J., Janoušková O., Scholtz V., Holada K. (2011). Inactivation of prions using electrical DC discharges at atmospheric pressure and ambient temperature. Plasma Process. Polym..

[30] Baxter H.C., Campbell G.A., Whittaker A.G., Jones A.C., Aitken A., Simpson A.H., Casey M., Bountiff L., Gibbard L., Baxter R.L. (2005). Elimination of transmissible spongiform encephalopathy infectivity and decontamination of surgical instruments by using radio-frequency gas-plasma treatment. J. Gen. Virol..

[31] Von Keudell A., Awakowicz P., Benedikt J., Raballand V., Yanguas-Gil A., Opretzka J., Flötgen C., Reuter R., Byelykh L., Halfmann H. (2010). Inactivation of bacteria and biomolecules by low-pressure plasma discharges. Plasma Process. Polym..

[32] Roth S., Feichtinger J., Hertel C. (2010). Characterization of *Bacillus subtilis* spore inactivation in low-pressure, low-temperature gas plasma sterilization processes. J. Appl. Microbiol..

[33] Sakudo A., Higa M., Maeda K., Shimizu N., Imanishi Y., Shintani H. (2013). Sterilization mechanism of nitrogen gas plasma: Induction of secondary structural change in protein. Microbiol. Immunol..

[34] Pattison I.H. (1966). The relative susceptibility of sheep, goats and mice to two types of the goat scrapie agent. Res. Vet. Sci..

[35] Prusiner S.B., Cochran S.P., Groth D.F., Downey D.E., Bowman K.A., Martinez H.M. (1982). Measurement of the scrapie agent using an incubation time interval assay. Ann. Neurol..

[36] Derkinderen P. (2019). Could it be that neurodegenerative diseases are infectious?. Rev. Neurol..

[37] Kovacs G.G. (2019). Molecular pathology of neurodegenerative diseases: Principles and practice. J. Clin. Pathol..

[38] Jaunmuktane Z., Brandner S. (2019). Invited Review: The role of prion-like mechanisms in neurodegenerative diseases. Neuropathol. Appl. Neurobiol..

[39] Prusiner S.B. (2012). Cell biology. A unifying role for prions in neurodegenerative diseases. Science.

[40] Marin-Moreno A., Aguilar-Calvo P., Moudjou M., Espinosa J.C., Beringue V., Torres J.M. (2019). Thermostability as a highly dependent prion strain feature. Sci. Rep..

[41] Murayama Y., Yoshioka M., Yokoyama T., Iwamaru Y., Imamura M., Masujin K., Yoshiba S., Mohri S. (2007). Efficient in vitro amplification of a mouse-adapted scrapie prion protein. Neurosci. Lett..

[42] Sakudo A., Lee D.C., Saeki K., Matsumoto Y., Itohara S., Onodera T. (2003). Tumor necrosis factor attenuates prion protein-deficient neuronal cell death by increases in anti-apoptotic Bcl-2 family proteins. Biochem. Biophys. Res. Commun..

[43] Sakudo A., Onodera T., Ikuta K. (2008). PrP^Sc^ level and incubation time in a transgenic mouse model expressing Borna disease virus phosphoprotein after intracerebral prion infection. Neurosci. Lett..

[44] Inoue Y., Yamakawa Y., Sakudo A., Kinumi T., Nakamura Y., Matsumoto Y., Saeki K., Kamiyama T., Onodera T., Nishijima M. (2005). Infection route-independent accumulation of splenic abnormal prion protein. Jpn. J. Infect. Dis..

